# An interoperable and reusable research environment for digital medicine

**DOI:** 10.3389/fdgth.2026.1943167

**Published:** 2026-10-28

**Authors:** Severin Kohler, Falk Meyer-Eschenbach, Leon Schüßler, Maximilian Meixner, Florian Kärcher, Andreas Kling, Vivien Schubert, Dirk Meyer zum Büschenfelde, Michael Marschollek, Roland Eils

**Affiliations:** 1 Center of Digital Health, Berlin Institute of Health at Charité—Universitätsmedizin Berlin, Berlin, Germany; 2 Department of Mathematics and Computer Science, Freie Universität Berlin, Berlin, Germany; 3 Health Data Science Unit, Heidelberg University Hospital and BioQuant, Heidelberg, Germany; 4 Peter L. Reichertz Institute for Medical Informatics of TU Braunschweig and Hannover Medical School, Hannover, Germany; 5 Institute of Medical Informatics, Charité—Universitätsmedizin Berlin, Berlin, Germany; 6 Clinical Study Center, Berlin Institute of Health at Charité—Universitätsmedizin Berlin, Berlin, Germany; 7 Hochschule für Technik und Wirtschaft Berlin University of Applied Sciences, Berlin, Germany; 8 Intelligent Medicine Institute, Fudan University, Shanghai, China

**Keywords:** clinical information models, EHR, FHIR, interoperability, openEHR

## Abstract

Clinical research infrastructures remain fragmented and project-specific, with semantic inconsistency accumulating across studies and constantly increasing “interoperability debt”. Repeated transformations between heterogeneous standards cause semantic drift and information loss. Prospective studies require duplicate data entry across clinical and research systems, increasing workload and introducing inconsistencies. Project-driven data models accumulate technical debt that undermines the sustainability and maintainability of research infrastructure across studies and funding cycles. Here, we explore how a reusable research environment can be built, and whether governing data semantics at the persistence layer provides a foundation that outlasts individual projects. We present an environment addressing these deficiencies at the architectural level. An openEHR-based clinical data repository with governed archetypes serves as the canonical persistence layer, integrating retrospective EHR data, prospective study data, patient-reported outcomes, and wearable sensor streams under a shared semantic model. Prospective data is captured directly through openEHR-based forms, which removes the separate capture system for study variables, while duplicate documentation with a hospital information system that does not run on openEHR remains. Reusable transformation pipelines support bidirectional FHIR interoperability and OMOP-based analytics. Federation nodes at three institutions were tested with cohort count queries across sites and are not yet in routine use. Deployed at Charité university medical center across five studies covering approximately 16,000 patients and 7.9 million data points, the environment shows that one governed repository can be reused across successive studies from different clinical domains within an existing healthcare IT landscape. Of its 125 archetypes, 57 are shared between at least two studies. Sustainability beyond these five studies and scaling beyond one institution remain to be shown. All tooling is publicly available and provided as a deployable configuration for institutional adoption.

## 1 Introduction

The rapid digitalization of health care has transformed medical research into a data-intensive enterprise (1). Secondary use depends on routine care data being accessible and of sufficient quality. Despite widespread adoption of electronic health records (EHRs), the data remains heterogeneous in structure and semantics, limiting its utility for research (1, 2). Each research project brings its own domain, requirements, and limited funding phase. Infrastructure is therefore often built as project-specific instances within the landscape of healthcare systems, with sustainability planning rarely formalized beyond the funding period (3). The questions research can address are, in turn, constrained by what these systems can deliver. These limitations are systemic barriers to sustainability, not mere technical inconveniences. Clinical data is longitudinal and shared across studies, with recurring patient records over time, and infrastructure must be longitudinal and shared in the same way. Failing to do so results in compounding costs instead of institutional amortization.

The structural problems recur across the literature. Data quality is limited at the source by the constraints of routine clinical documentation (2, 4). Fragmented research infrastructures and locally adapted implementations prevent reuse, leading to parallel silos that cannot easily interoperate (5, 6). The same data is transformed redundantly into different target representations across projects, each lossy (7, 8) and requiring its own maintenance (9, 10). Routine clinical documentation and prospective research data capture stay misaligned, forcing duplicate entry into study-specific forms (11).

These pressures are amplified by the trajectory of clinical research itself. Multi-site studies extend the same problems across institutions rather than within them, raising the coordination and harmonization burden (12, 13). Artificial intelligence approaches add demand for large, semantically consistent longitudinal datasets that fragmented project infrastructure cannot reliably provide (14). Emerging regulatory frameworks such as the European Health Data Space (EHDS) introduce further requirements on the underlying infrastructure (15). As complexity grows, these costs accumulate, limiting both the scope of research that can be undertaken and the efficiency with which it can be done.

The requirements for a research environment that can address these challenges are complex and must integrate the structural and architectural dimensions outlined above. This study derives such a set of requirements through a hybrid approach combining literature and implementation experience at Charité—Universitätsmedizin Berlin, and presents an architecture that satisfies them. The architecture realizes them by maintaining clinical data models, terminologies, and pipelines as governed infrastructure shared across studies instead of artifacts rebuilt within each project. It is deployed at Charité and evaluated across five research studies, with archetype reuse and cross-study integration used as primary indicators of whether the infrastructure is reused across studies instead of rebuilt.

## 2 Methodology

### 2.1 Study design

This work proceeds in four steps. First, architectural requirements are derived from the literature and from implementation experience at Charité, reported in the remainder of this section. Second, a standard independent reference architecture is designed against these requirements, and a persistence standard is selected for its instantiation. The reference architecture and its instantiation are the designed artifact of this work and are reported in Sections 3.1, 3.2. Third, the instantiated architecture is implemented at Charité, reported in Section 3.3. Fourth, the implementation is evaluated across five research studies. Archetype reuse across studies, the number of transformation pipelines shared between studies, and the adaptation effort of each new study serve as indicators of whether the infrastructure is reused and not rebuilt. These observations are reported in Section 3.3 and taken up in the Discussion.

### 2.2 Derivation of architectural requirements

In the course of developing and deploying the repository, recurring structural limitations became evident across independent research initiatives and interoperability implementations. The team collected them retrospectively from the integration work for the five studies and the institutional data lake. Exchange with other institutions and at conferences showed that other sites face the same problems. These observations were grouped into the seven structural problem classes of Table 1, and for each class the literature was searched for reports of the same problem from other settings, so that no class rests on this deployment alone. Each class was then translated into one architectural requirement, the property of the environment that removes the cause of the problem, and the set was agreed within the team.

**Table 1:** Structural challenges affecting the secondary use of EHR data.

Structural challenge	Manifestation in secondary use
Data quality	Limited fitness of EHR data for reuse, including missing values, inconsistent coding, and variable documentation practices
Fragmented research infrastructures	Project-specific research infrastructures with limited reuse of pipelines, mappings, and supporting tooling
Redundant data transformations	Repeated and potentially lossy transformations between heterogeneous standards and local data representations
Clinical-research documentation misalignment	Duplicate data entry in prospective research and missing study-specific fields not captured in routine clinical documentation
Governance and sustainability	Lack of formal governance and long-term lifecycle management for data models, mappings, and repository operations
Data complexity and cross-site interoperability	Growing data complexity (multi-modal data) and increasing requirements for cross-system and cross-site interoperability
Technical lock-in and limited accessibility	Restricted data access due to vendor-specific systems and siloed infrastructures

These problem classes are structural deficiencies. The quality and fitness of EHR data for secondary use is limited due to heterogeneous documentation practices at the point of capture, incomplete datasets, inconsistent coding, and fragmented data sources across clinical systems (2, 4). Such data must be preprocessed before use, and where this happens through project-specific approaches rather than a shared, governed basis, the same source data can yield divergent results. Applied to a single dataset, implementations of one Sepsis-3 definition produced detection rates ranging from 3.4% to 65.2%, a spread attributable to preprocessing, not the data itself (16). At the same time, fragmented research infrastructures and locally adapted implementations prevent reuse, leading to parallel silos that cannot easily interoperate (5, 6).

Data quality and fragmented research infrastructures, the first two classes, degrade the data at its source, before any study is designed. Variable documentation at the point of capture, incomplete records, and inconsistent coding mean that the same clinical fact is often recorded differently or not at all across the source systems (2, 4). Because access to these sources is rebuilt separately for each project, this integration effort is rarely reused and accumulates as isolated silos (5, 6).

The per-project rebuilding of access and integration leads to redundant transformations between heterogeneous representations. Each project reconstructs its own transformation logic, increasing maintenance overhead and introducing the risk of semantic loss. Consequently, healthcare organizations often invest significant resources in developing and maintaining custom Extract-Transform-Load (ETL) pipelines (9, 10, 17). Adopting a standard such as FHIR, openEHR, or OMOP shifts this cost without removing it. Each pipeline must be built and continuously revalidated as standards evolve and source systems change. These ongoing costs are rarely accounted for in project budgets, yet accumulate across the organization as a hidden cost of interoperability.

Misalignment between routine clinical documentation and prospective research data capture leads to duplicated effort, as data must be repeatedly transcribed across systems, increasing workload and the risk of errors (11). Additionally, research protocols often require study-specific fields that are not captured in routine clinical documentation, necessitating separate manual data collection. In two studies of the deployment described in this paper, CAEHR-C and CALM-QE, this resulted in 27 and 17 prospective forms, requiring manual re-entry of information already available and additional fields introduced for research variables.

Differences in how sites govern their clinical data models, terminology mappings and ETL pipelines let semantic heterogeneity grow between sites and over time (18, 19). Sustaining these components requires coordinated lifecycle management, spanning model versioning and terminology curation together with repository operations, commitment that is rarely formalized (20, 21), and infrastructure built around specific projects rarely has sustainability formalized beyond the initial funding period (3).

The volume and heterogeneity of data integrated in clinical research continues to grow, driven by the adoption of multi-modal data sources and the increasing amount of multi-site studies, which further amplify interoperability requirements and coordination complexity (12–14).

Vendor-specific EHR systems and proprietary data formats frequently prevent access to clinical data that would otherwise be valuable for secondary use (9, 22, 23). Healthcare institutions typically operate either monolithic single-vendor platforms, which risk locking data into a closed ecosystem, or fragmented best-of-breed deployments, where data is spread across disparate systems with little clarity over where it resides. In either case, data captured in routine care remains locked within institutional or vendor boundaries, making it unavailable for research and quality monitoring as well as cross-institutional analysis (2, 22, 24).

These structural challenges are not mutually exclusive and form a reinforcing cycle in which each amplifies the others. As an example, fragmented infrastructures drive redundant transformations, introducing semantic loss that compounds existing data quality issues. Technical lock-in restricts access to source data, further limiting the ability to validate and correct records downstream. Governance deficits allow these problems to accumulate silently, while growing data complexity and multi-site requirements amplify every layer of this cycle, collectively undermining the FAIR principles that effective secondary use of clinical data depends upon (25).

These structural patterns are observable at Charité—Universitätsmedizin Berlin, where data integration spans many source systems, project-specific transformations, and governance arrangements that have grown across independent initiatives and projects. Data from more than 16 source systems are ingested mostly daily, depending on project needs and technical feasibility, into a Health Data Lake organized across four layers (26). The raw layer mirrors data of source systems in a pseudonymized way for several hundred tables. Building on top, the prepared layer consolidates these into around 65 use-case-independent assets. As example the 56 COPRA6 intensive care tables are reduced to 12 combined and simplified tables. By design, this consolidation operates at the structural rather than the semantic level, leaving semantic harmonization to downstream projects.

The same pattern is visible in data extraction. Projects can request an on-boarding to work directly on a data separation in the data lake, while others submit requests for CSV-based data extractions to the Service Unit Data Science, which served 224 simple and 29 advanced requests over the last two years. Advanced requests require extensions of the script to enable custom cohorts, time windows, schema mapping, preprocessing, extractions or the ingestion of further source tables into the data lake. FHIR resources aligned with the Medical Informatics Initiative core dataset exist in the interpreted layer but serve institutional reporting. No project, as of now, has requested extraction from them, so pipeline extension follows request history, not the semantic fitness of the representation. Projects may steward generically prepared assets themselves, but in practice this requires sustained domain-expert involvement that individual project funding rarely sustains, so few such assets are actively maintained. Each request is parameterized through a request-form individually, with semantic parameter selection left to each project through a list of available parameters instead of being resolved once in a governed layer.

### 2.3 Architectural principles for sustainable research infrastructures

In clinical infrastructures, lifecycle management of data models must be designed to span decades. While the underlying technical stack may evolve over time, the core data models and governance frameworks must remain stable and traceable across decades of maintenance. The real cost of infrastructure does not lie in building or adopting tools but in the maintenance tail that follows poor architectural decisions (27). Ad-hoc solutions postpone structural problems without actually resolving them, accumulating what Cunningham termed technical debt (28), the implicit future cost of choosing an expedient solution over a correct one. Clinical data is inherently longitudinal, patients are followed over years or decades, an architecture must be built around this reality. Where data models and their pipelines are not designed for reusability and long-term traceability from the outset, accumulated debt threatens not only operational efficiency but the scientific integrity and regulatory compliance of the systems that carry that data over a patient’s lifetime. Sustainable architecture is therefore a prerequisite for any system carrying clinical data over decades. The structural challenges identified in this chapter are not unique to clinical research infrastructure, they are well-recognized consequences of poor software and systems design. Established patterns such as separation of concerns, reuse, abstraction, and open interfaces exist precisely to address them, and form the guiding design principles of the architecture proposed here.

Poor primary use data quality should not justify equally poor secondary use infrastructure. Instead, it makes principled design more important. A well-governed secondary use architecture remains valuable independent of upstream data quality and positions the system to improve as that quality evolves. Integrating clinical data into a reusable, standards-based secondary use architecture can itself be seen as a first step toward standardizing EHR data, establishing the models and governance patterns that primary systems could progressively adopt (7). This is only achievable if the persistence layer itself imposes semantic discipline rather than deferring it to downstream consumers.

The inadequacy of current approaches is evident across both ends of the architectural spectrum. Data warehouses provide structure through fixed schemas but lack semantic governance. This is particularly evident in i2b2, where the fixed schema cannot faithfully represent logically connected clinical events (8). Data lakes offer flexible storage at scale but abandon structural discipline, devolving without enforcement into data swamps of inconsistent provenance. A clinical data repository (CDR)-based architecture grounded in clinical standards addresses what both approaches lack. It provides a governed semantic model in which clinical data meaning is explicit and standardized. Where a CDR serves as the governed semantic core, a warehouse becomes a legitimate analytical extension and a data lake a valid staging ground for data not yet standardized. The problem arises only when a warehouse or data lake substitutes for a controlled semantic data model, leading to the architectural debt that disciplined design seeks to avoid.

### 2.4 openEHR archetypes and templates

openEHR expresses clinical information models as archetypes. Its two-layer approach separates the technical reference model from clinical knowledge, which lowers the barrier for reaching clinical consensus. Unlike project-driven models, which capture only study-required variables, archetypes follow an inclusive modeling approach. Instead of scoping the model to a specific study context, an archetype aims to represent all clinically relevant information within a concept. Archetypes are developed in an international community and published on the Clinical Knowledge Manager (CKM), an open library covering around 900 models today (29). Project-specific data requirements are addressed through templates, which constrain and combine archetypes to a study context without modifying the underlying models. Projects can adopt published archetypes and contribute back to them under the same governance framework that governs the published library.

## 3 Results

### 3.1 Reference architecture

The reference architecture answers the structural challenges of Section 2 with the requirements listed in Table 2, built around a semantically governed clinical data repository as the persistence layer, as illustrated in Figure 1. The CDR occupies the center of the architecture as the single governed source from which all downstream representations are derived. It is required to maintain a longitudinal, patient-centric record and to enforce semantic consistency at the model level, shifting quality assurance upstream and away from downstream consumers. Data is structured according to Clinical Information Models (CIMs), developed with clinical consensus to reflect real-world documentation practice instead of technical convenience. A CIM specifies a health and care concept as defined data elements with their data types, value sets and constraints (30). Two properties let a pipeline from the CDR be written and maintained once per model. The model is governed and shared, ideally internationally, so every study and site that uses it maps against the same definition. Its elements carry defined meaning, types and value sets, so the pipeline can be written against the model.

**Table 2:** Architectural requirements addressing the structural challenges affecting secondary use of EHR data.

Structural challenge	Architectural requirement
Data quality	Persistence layer enforcing semantic consistency at the model level
Fragmented research infrastructures	Centralized multi-study repository as shared governed infrastructure
Redundant data transformations	Reusable transformation pipelines and mapping specifications derived from a canonical representation
Clinical-research documentation misalignment	CDR-compatible forms integrating clinical and research data capture in a single workflow
Governance and sustainability	Formal lifecycle management for clinical information models and transformation pipelines
Data complexity and cross-site interoperability	Extensible semantic modeling with a shared governance layer for multi-site deployment
Technical lock-in and limited accessibility	Open specifications and vendor-independent interfaces

**Figure 1:**
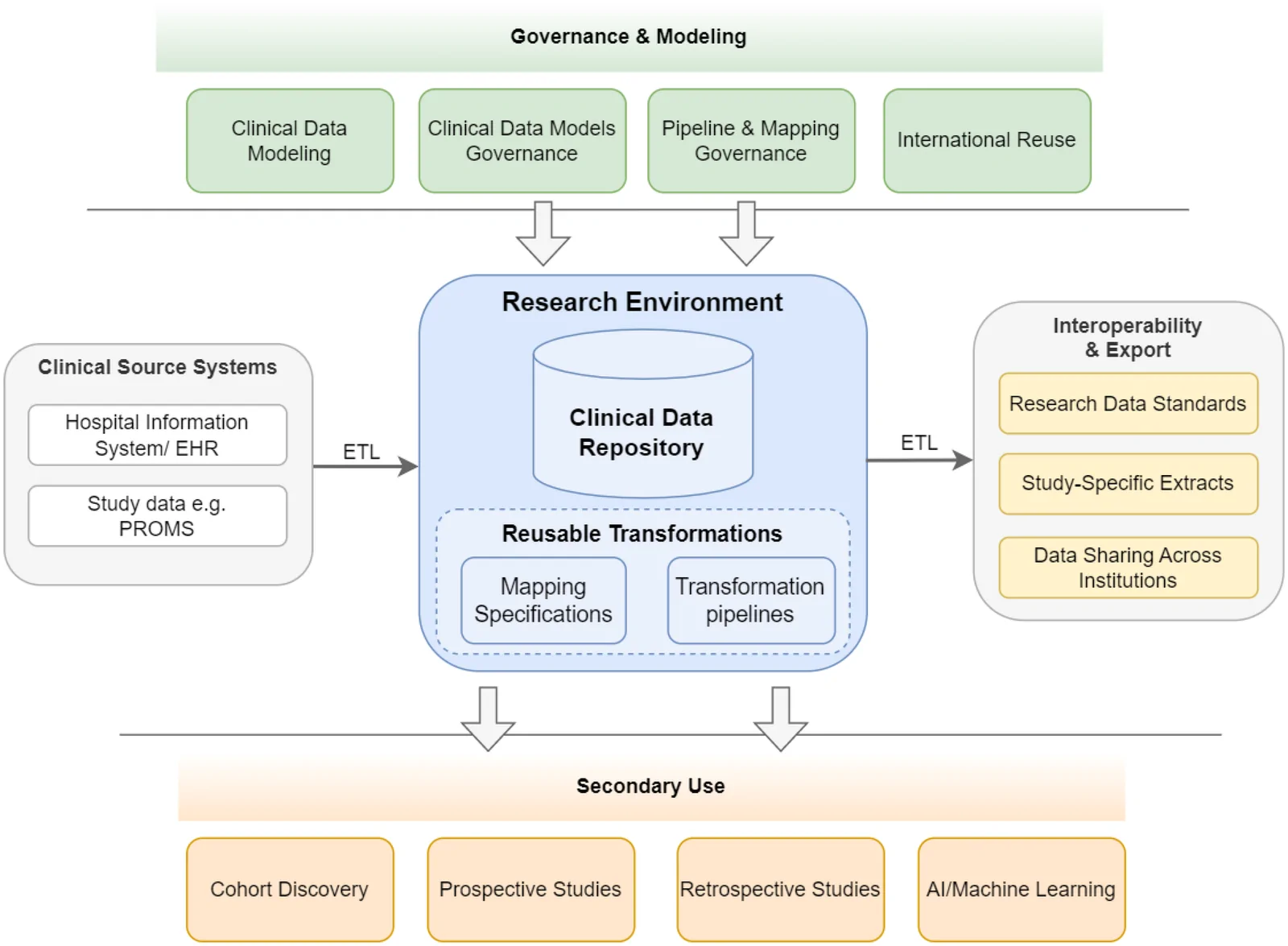
Reference architecture for integrated clinical and research data management. The architecture centers on a clinical data repository that enables data persistence, reusable transformation pipelines, and interoperability with external research data models and clinical systems. Mapping specifications are shown as a separate artifact. Both the pipelines and the mapping specifications are reused across studies and sites.

A central CDR reduces fragmentation by providing a single governed source to which study data is collected and from which project-specific representations are derived. A transformation pipeline has two parts. The engine implements a target standard once and does not change from study to study. The mapping specifications define how the elements and value sets of a model are projected onto the target standard, are written per model and apply to every study that uses it. Figure 1 therefore shows the specifications as an artifact of their own next to the pipelines.

Both the CIMs and the pipelines built on them require governance that extends beyond individual project funding cycles. Without this, semantic divergence accumulates across projects and pipeline inconsistencies go undetected, undermining the stability that longitudinal data persistence requires. In practice this means that models and mappings are versioned and that every mapping records the model version and the target standard version it was written for. Neither is released without review by designated reviewers, either in a community process or within the institution. The CDR must further operate through open, vendor-independent interfaces to prevent proprietary lock-in at the persistence layer. Open specifications means that the interfaces of the repository are published and free to implement, for example the persistence model, the query language and the access interface. No vendor specific interface then sits between the data and its consumers. CIMs need to be extensible to accommodate growing data complexity of modern and future research.

The CIMs used to persist data in the CDR should be designed to support two complementary data capture pathways, reuse of data already captured in routine clinical care, and study-specific forms that can be integrated directly into primary use systems. Such a form is generated from the clinical information model of the study, validated against it on commit and stored in the repository. Together these pathways eliminate the need for parallel electronic data capture (EDC) systems and reduce duplicate documentation to the minimum unavoidable by study protocol.

Each of these properties corresponds directly to one of the structural challenges identified in Section 2, as summarized in Table 2. The following section describes the instantiation of this architecture using openEHR as the persistence standard, as motivated in Section 2.

### 3.2 Proposed architecture instantiation

A sustainable research environment therefore requires a CDR persistence standard that is longitudinal, patient-centric, and semantically governed. Neither FHIR, optimized for exchange, nor OMOP, optimized for analytical queries (31), were designed to fulfill these persistence requirements. Both rely on Terminology-Driven Concept Identification (TDCI) (7, 32, 33). In a terminology-driven model, the clinical concept is identified by a terminology code placed in a generic structure. A systolic blood pressure in OMOP is a Measurement row whose meaning comes from the concept identifier. In a model-driven approach, the concept is identified by the model itself. The openEHR blood pressure archetype has an element for systolic pressure, and its meaning is fixed by the archetype, with terminology bindings added on top (33). TDCI offers flexibility and low upfront modeling cost, properties well suited to data exchange and ad-hoc analytics, but whose generic structures require either project-specific extensions, fragmentation or the omission of data.

Of the 16 elements of the Problem/Diagnosis archetype, 4 map directly to condition_occurrence in OMOP and 11 need auxiliary records linked through fact_relationship, a construct that OMOP tooling rarely queries. The clinically recognised time has no OMOP field or concept at all (33). The pattern holds across the 198 archetypes of the OMOCL library (33). FHIR keeps such elements through extensions, which are defined per profile, for example for the needs of one project. The Procedure archetype has 8 elements with no counterpart in the Procedure resource that need them (32). Around 35 percent of the fields in FHIR projects are extensions and four out of five of these are not standardised, so the same element is carried differently from project to project (32, 34). This is not a weakness of FHIR or OMOP. Their generic structures let one schema serve many domains and institutions, and a representation that drops an element not needed for a defined exchange or a predefined analysis remains fit for that purpose. The loss matters where the generic form is the persistence layer and the use of the data is not fixed in advance, as in AI model training on complete records, for example for multi disease prediction. An element that was never represented cannot be recovered later, and the companion evaluation found that this raises the risk of subtle and undetectable errors in downstream analyses and study conclusions (33).

Model-Driven Concept Identification (MDCI), by contrast, encodes clinical meaning explicitly in the model. It requires more governance effort upfront and needs alignment between institutions before a model is adopted widely. In return it yields a stable, reusable semantic foundation that amortizes across decades of data, the trade-off a sustainable architecture must favor for long-term persistence. FHIR is the stronger standard for exchange and OMOP for analytics (31–33). openEHR is designed as a longitudinal EHR following an MDCI approach with an internationally governed model library, and is therefore the better fit for the persistence layer. A sustainable research environment must support other standards like FHIR and OMOP, so the CDR must provide transformation pathways to both. Figure 2 illustrates the resulting openEHR-based instantiation of the reference architecture.

**Figure 2:**
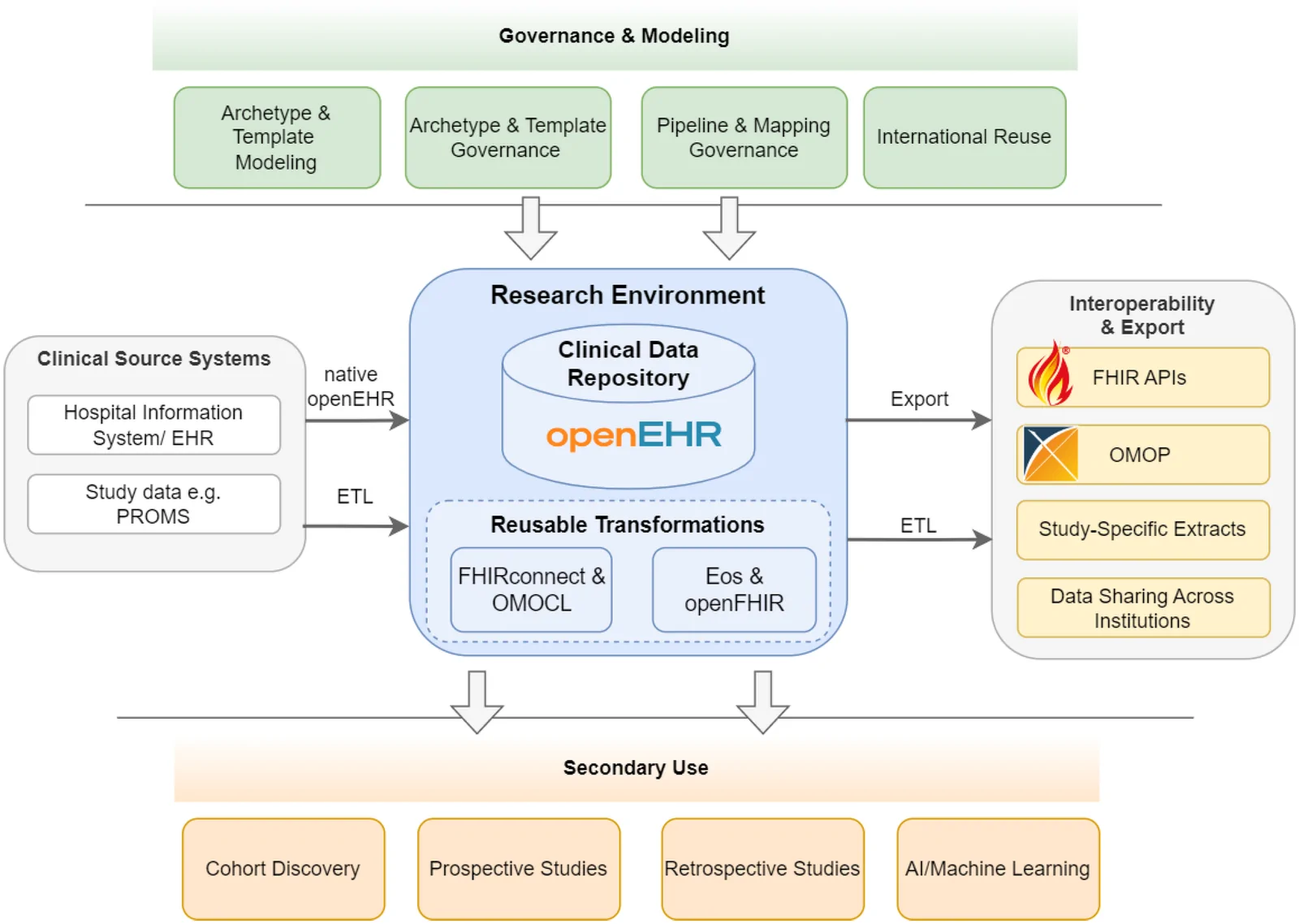
openEHR-based instantiation of the reference architecture. Archetypes and templates take the place of the CIMs, Eos and openFHIR are the engines, and the OMOCL and FHIRconnect mapping libraries are the mapping specifications.

openEHR satisfies the requirements of Table 2 as follows. Semantic consistency at the model level follows from archetypes carrying the clinical meaning of each element, so the representation of data persisted in the CDR is governed by the model and the governance process extends beyond any single project. This is one part of data quality (4). For prospectively captured data the template also enforces mandatory elements and value constraints at entry. For data taken over from source systems the model fixes the representation, but whether a recorded value was true or current for the patient remains a property of the source. A single CDR hosts isolated instances per study, so several studies share one governed repository. Pipelines defined on archetypes are reusable across all projects that share the same archetypes. With internationally governed archetypes this reuse extends across institutions. Two sites targeting the same output, such as OMOP, share one mapping if they use the archetype as intended. A local deviation can be expressed, if necessary, in the mapping language, not in the pipeline. This lowers the implementation effort for each new research project. The documented and scoped elements of an archetype make field identification during mapping unambiguous. Local and proprietary models developed without formal governance often lack this granularity and documentation. Distinct clinical concepts then collapse into shared fields, a conflation that propagates directly into downstream analytics.

Study-specific capture is supported through templates rendered as forms. Where the primary EHR runs natively on openEHR, routine clinical data flows into the CDR without transformation and study variables are captured in the clinical workflow, so both redundant conversion and duplicate documentation are avoided. Lifecycle management is provided by the governed versioning of archetypes, applied locally or, for internationally maintained archetypes, across institutions. The granularity and domain coverage of clinical models must grow as research hypotheses evolve. Archetypes accommodate this by introducing new archetypes or extending existing ones. Because clinical knowledge is separated from the technical layer, this semantic scaling requires no schema change to the repository. In a conventional database-backed model such a change is unavoidable. Static, project-specific designs make such extension more fragile and the change process harder to control. Internationally governed archetypes further provide the shared governance layer that multi-site deployment requires, since sites using the same archetypes persist comparable data. The specification is open and vendor independent, so the persistence layer introduces no proprietary lock-in as data volumes and system dependencies grow.

### 3.3 System architecture and deployment

We implemented the proposed architecture at Charité - Universitätsmedizin Berlin. Not all aspects of the architecture could be implemented, since Charité does not use openEHR natively in its Hospital Information System (HIS). The overall architecture of the research environment is illustrated in Figure 3.

**Figure 3:**
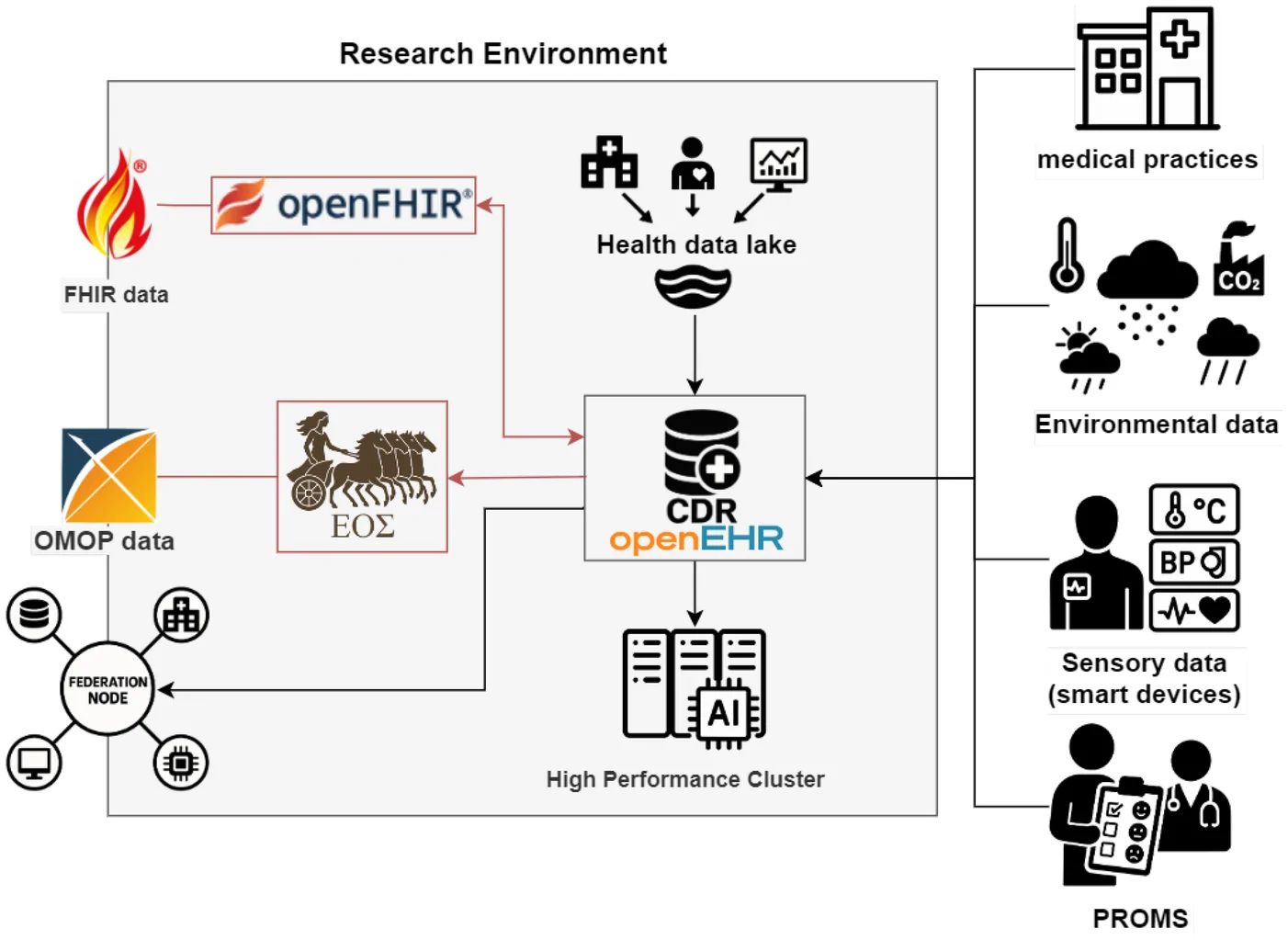
Architecture of the implemented research environment at Charité. Retrospective clinical data are ingested from the institutional Health Data Lake via ETL pipelines into the openEHR-based clinical data repository (CDR). Prospective research data, patient-reported outcomes (PROMs), and wearable sensor data are captured through standardized interfaces directly into the CDR. From the CDR, reusable transformation pipelines project data into FHIR and OMOP representations, while direct exports support analytical workflows on the high-performance computing cluster.

The core of the architecture is the CDR, which supports multiple isolated instances within a single repository. This reduces fragmentation and maintenance effort by providing each study with its own isolated environment while enabling cross-instance queries where access rights permit. In some studies a FHIR server deployed as part of the CDR adds a second layer that keeps the study pseudonym out of the openEHR record and provides a standard identity API without custom development. Its Patient resource carries the study pseudonym and the openEHR record only references it. Other studies carry the study pseudonym directly in the openEHR record, depending on their requirements. The data stored in openEHR only contains anonymized and pseudonymized data. Each dataset is integrated based on openEHR archetypes.

Retrospective integration was achieved for around 190,000 clinical records across five studies via ETL pipelines from the institutional Health Data Lake (26), which consolidates heterogeneous source systems at Charité into the CDR using governed archetypes. Data preparation for these pipelines deduplicated records carried by several source systems and bound local codes to terminologies before commit. Pseudonymization is layered across both systems. First-order pseudonyms are generated when data is ingested into the lake, using SHA-256 hashing of patient identifiers. For each project-specific extraction from the lake, second-order pseudonyms are derived by hashing the first-order pseudonym together with a project acronym and a project-specific salt. The openEHR CDR contains project-scoped, second-order pseudonymized records. This second-order pseudonym is the study pseudonym referred to above.

Prospective data capture was implemented without transformation, all clinically documented data, including patient-reported outcomes and outpatient data from studies operating outside Charité, was entered directly into the CDR through openEHR-based forms. A form is generated from the study template and rendered in the browser by a form component. Each entry is validated against the template on commit and stored as a versioned composition with committer and timestamp, so study variables are captured in the same governed models as the retrospective data. Where native openEHR systems are in place, this approach additionally eliminates duplicate documentation entirely. In the present deployment, study-specific data collection required no ETL step, but parallel documentation with the hospital information system remains. For data sources without interoperability support, including wearable devices and environmental sensors, dedicated ETL pipelines handle ingestion. All ETL pipelines map their input to the governed openEHR archetypes of the CDR. This mapping, not the ingestion itself, yields a unified semantic layer spanning clinical, patient-reported, and real-world sensor data across more than 900 prospectively enrolled patients and five studies within a governed repository.

Three reusable transformation ETL pipelines were implemented for all downstream representations. FHIR-based interoperability is supported bidirectionally via FHIRconnect (32), enabling both export to external systems and import of FHIR-structured data into the CDR. Analytical workflows are supported through OMOP export via Eos (7) and direct openEHR data exports to analytical environments such as high-performance computing clusters for AI research.

A mapping is defined once per archetype and applies to every study that uses the archetype. The Problem/Diagnosis archetype, for example, appears in 14 templates of four studies. One OMOCL mapping and one FHIRconnect model mapping cover all of them, and neither was written for any of these studies. Of the 57 archetypes shared between at least two studies, 34 can be exported to OMOP and 24 to FHIR with the mappings that ship in the shared libraries. No study specific mappings have been added to the deployed pipelines, since no study has requested an export so far, and the pipelines have therefore not run in production at Charité.

For OMOP, OMOCL holds one mapping per archetype (7, 33). Terminology mappings are part of the same file, as concept maps that translate archetype codes to OMOP concepts (33). An archetype without a mapping would receive one, preferably in the shared library. A local deviation would be an edit to the mapping file, which has not been reported in the community so far. For FHIR, model mappings form the base layer, written once per archetype and resource, and extension mappings cover the profiles of a study and the deviations of a site. Value set translations are written inline in the mapping as pairs of openEHR and FHIR codes. In the current library, 365 of 542 path mappings (67.3%) sit in the profile independent model mappings, which quantifies their reuse (32). A new study therefore needs a template and, for archetypes not yet covered, mappings in OMOCL and FHIRconnect. In both pipelines the engine, the existing mappings and the data type transformations stay untouched. Provenance is kept in the CDR, where each composition is versioned and records committer and timestamp, and FHIRconnect writes the originating system and source record of imported FHIR data into the feeder audit. The OMOP export carries no provenance of its own. Its lineage is determined by the CDR snapshot and the versions of pipeline, mappings and vocabulary that produced it, and a standardized provenance record is being developed for both OMOCL and FHIRconnect (32).

Both mapping libraries consist of declarative mapping files in a documented mapping language, one file per archetype, and are maintained in public repositories. Each mapping file carries a header defined by the mapping language. It records the version of the mapping, the archetype it maps and the version of the target standard, OMOP or FHIR. In FHIRconnect it also records the archetype revision and the target profile (7, 32). A new archetype revision or standard version therefore yields a new mapping version and not a new pipeline. Changes are peer reviewed in the team and by the respective community on the open repository and covered by unit tests in the engine repositories. This process was exercised as part of HiGHmed on the mappings of the Medical Informatics Initiative core data set to FHIR and, initially, of the German Corona Consensus data set to OMOP.

All pipelines and supporting tooling were developed as part of this work and are publicly available as open-source software, including a cohort explorer for interactive cohort definition, Archetype Query Language (AQL) federation nodes enabling cross-site querying across distributed CDR instances, an openEHR record export tool, and a synthetic test data generator for pipeline validation. Federation nodes are currently deployed as validation instances at Charité, Vall d’Hebron University Hospital, and Karolinska Institutet. The federation node sits in front of the multi-study repository and returns cohort counts for patients who have consented to such queries. A federated query can run across all study instances inside the CDR. A query is submitted to the nodes of the participating sites, each node executes the AQL locally and returns the result. The output in the test was restricted to cohort counts for privacy reasons. Finer control is possible, for example by exporting a consented subset of records into a separate instance. Cohort count queries across the three validation instances have been tested successfully but have not yet been used in a live study. The CDR is one of several available implementations of the openEHR specification, open source and commercial. Forms, pipelines and queries reach it only through the openEHR REST API and AQL, so another implementation could take its place. The portable components of the environment, comprising an openEHR CDR, FHIR server, OMOP database, openFHIR, AQL federation node, cohort explorer, Eos, and export tooling, are provided as a deployable Helm chart and Docker Compose configuration, enabling out-of-the-box instantiation without manual integration overhead, open-source on GitHub at https://github.com/AltarisV/OHS-Open-Health-Stack.

The data models are based on international archetypes or archetypes developed within the HiGHmed community (21), governed through the respective CKM. Of the 125 unique archetypes used, 86 (68.8%) are drawn from the internationally governed library and 39 (31.2%) are locally governed. The majority of local archetypes address genuine gaps in the international library at the time of modeling, covering clinical scoring instruments and pulmonology-specific concepts not yet represented in the international library. These archetypes underwent HiGHmed CKM review or internal review and have been published to the HiGHmed CKM, with targeted submissions to the international CKM ongoing. The archetypes and templates of each study are available on the HiGHmed CKM under CALM-QE, CAEHR-C, ACRIBIS, HiGHmed-UCC and ETL-AI. ACRIBIS builds largely on the CAEHR-C models, and its own entry holds only the archetypes of its follow-up questionnaire. Not everything is published there, since some models are still in local refinement before review and some reflect local requirements of the hospital. The remainder comprises demographics models and German healthcare context-specific models, alongside a subset of legacy models from an earlier project phase that predate formal governance and are candidates for revision. Across the five studies, 57 of 125 archetypes (45.6%) were shared between at least two studies, with per-study and pairwise reuse detailed in Figure 4.

**Figure 4:**
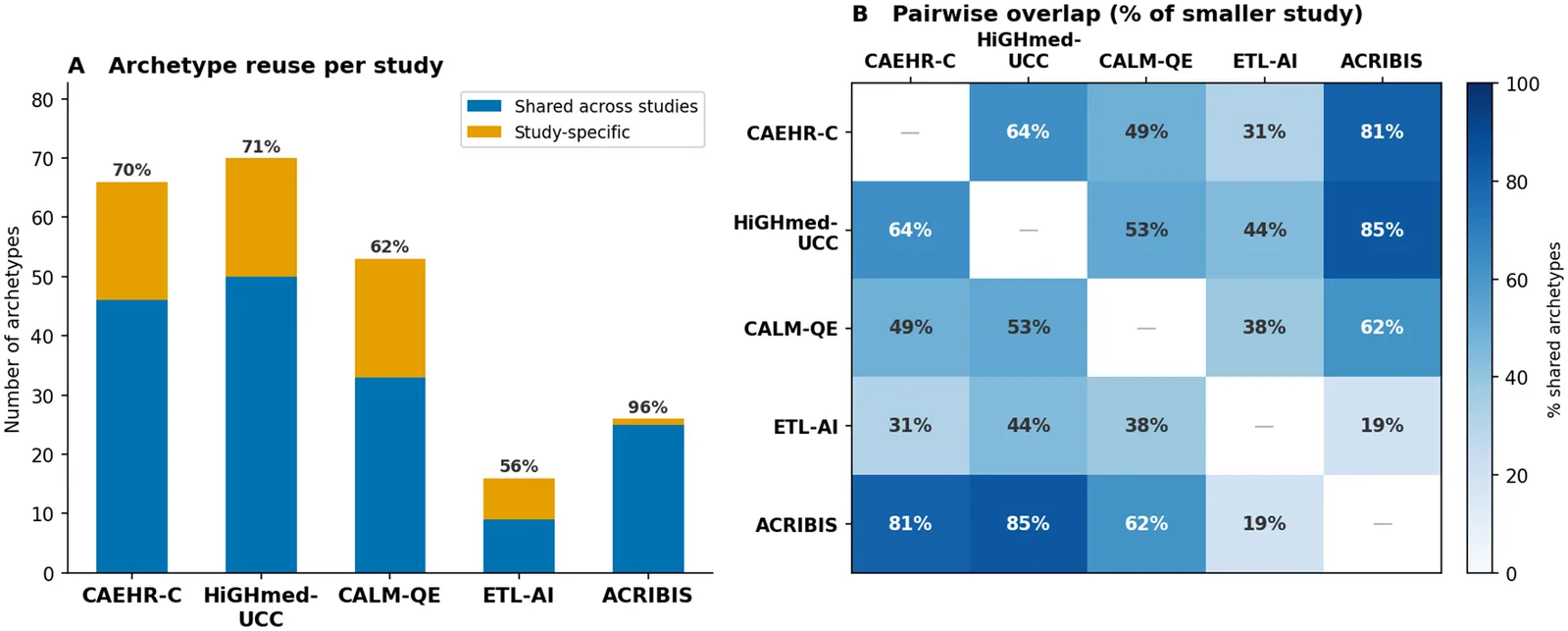
Archetype reuse across studies. **(A)** Total number of archetypes per study, stratified by shared (blue) and study-specific (orange) archetypes, with the percentage of shared archetypes indicated above each bar. **(B)** Pairwise archetype overlap between studies, expressed as the percentage of archetypes in the smaller study that are also present in the larger study.

The deployment matured across the successive studies listed in Table 3. The team observed that each new study took less time to onboard than its predecessor, regardless of its clinical domain. This onboarding time was not measured and remains an observation. The reasons are the reused components. Every study writes into the CDR through the same API, wearable and sensor data enter through the same ingestion route, the form and patient-reported outcome components were built once, and the experience of the team grew with each study.

**Table 3:** Studies conducted on the research environment.

Study	Clinical focus	Design	Data types
CALM-QE	Pulmonology	Prospective, cross-site	Outpatient care data, PROMs, wearables, environmental data
CAEHR-C	Cardiovascular disease	Prospective and retrospective, cross-site	Clinical, outpatient care data, wearable sensor data, PROMs
ACRIBIS	Cardiovascular risk assessment	Prospective, cross-site	Clinical, biosignals (ECG), PROMs
HiGHmed-UCC	Chronic heart failure	Prospective, cross-site	Clinical, outpatient care data, wearable sensor data
ETL-AI	Infection control	Retrospective	Clinical

Cross-site refers to the study design. None of the studies used the federation nodes.

What was measured is the origin of the artifacts of each study, listed in Table 4 in the order in which the studies came onto the platform. Without a mapping written for any study, the shared OMOCL and FHIRconnect libraries already covered 46 to 71 percent of each study’s archetypes for OMOP and 27 to 54 percent for FHIR. Supplementary Table S1 gives the share each study would still have had to write. ACRIBIS, in a domain already covered, found 25 of its 26 archetypes on the platform, modelled none and took both templates from CAEHR-C. Forms were imported from earlier studies and adapted where a similar one existed. CALM-QE needed more new artifacts again, 21 archetypes not used before and nine modelled locally, because pulmonology was a new domain on the platform. The number of artifacts still to produce therefore fell with the coverage of a clinical domain on the platform, while the reused components shortened onboarding regardless of domain.

**Table 4:** Origin of the templates and archetypes of each study, in the order in which the studies came onto the platform.

Study	Templates	Templates adapted from an earlier study	Archetypes from the CKM	Archetypes from an earlier study	Archetypes modelled locally
HiGHmed-UCC	15	0	53	0	17
CAEHR-C	17	7	18	42	6
ETL-AI	3	0	2	7	7
CALM-QE	12	4	12	32	9
ACRIBIS	2	2	1	25	0

Templates adapted from an earlier study were copied from that study and changed. Implementation time was not recorded.

The transition from cardiovascular (CAEHR-C, ACRIBIS, HiGHmed-UCC) to pulmonology research (CALM-QE), for example, required changes at the archetype level only, with no modifications to the underlying infrastructure, a direct consequence of the two-layer openEHR separation of clinical knowledge from the technical reference model. The shared repository enabled queries across study instances, allowing data reuse at the technical level, without per-project transformation or infrastructure adaptation. The semantic consistency of the shared archetypes ensured that such queries operated on comparable representations regardless of the originating study, so that results from different studies could be interpreted jointly. The central repository further enabled real-time monitoring of data inflow and quality indicators across all active studies, replacing a previously manual process that lacked systematic detection of documentation inconsistencies. Distinct test and production environments, differentiated solely by endpoint configuration, supported parallel operation across study phases without infrastructure duplication.

Table 5 summarizes how each structural challenge in Table 1 is addressed by a corresponding architectural mechanism and its observed implementation outcome.

**Table 5:** Observed implementation outcomes of the proposed research repository architecture.

Structural challenge	Architectural mechanism	Observed implementation outcome
Data quality	Canonical openEHR-based persistence model	Unified granular dataset combining retrospective clinical data and prospective research capture, retrospective data persisted as recorded
Fragmented research infrastructures	Shared multi-study clinical data repository	Multiple studies deployed within a single governed repository environment
Redundant data transformations	Reusable transformation pipelines	Reusable pipelines defined and publicly available for FHIR and OMOP projections (7, 32), direct export tooling available for analytical environments
Clinical-research documentation misalignment	Archetype-based research forms aligned with clinical models	Single-source study documentation combining clinical and study variables, parallel documentation with the hospital information system remains
Governance and sustainability	Version-controlled archetypes and transformation pipelines	Stable semantic alignment maintained across institutional deployments
Data complexity and cross-site interoperability	Federation-ready deployment and multi-source ingestion architecture	Cohort count queries tested across three institutions, not yet in production
Technical lock-in and limited accessibility	Open standards and interoperable APIs	Vendor-independent data access through standardized interfaces

## 4 Discussion

This work derives architectural requirements for a sustainable research environment, proposes a CDR-based instantiation, and demonstrates its feasibility through deployment at Charité across five studies, 16,000 patients and 7.9 million data points. The architecture centers on an openEHR CDR, integrating heterogeneous data types including clinical records, PROMs, environmental and wearable sensor data. Governed data models form the basis from which reusable transformation pipelines derive standardized representations to FHIR and OMOP for downstream use. The architecture stack is accessible open-source as a deployable Helm chart and Docker Compose configuration. Institutions facing these structural challenges can adopt the architectural principles independently of the stack, or deploy the environment, with the potential to reduce the cost of building and maintaining research infrastructure across projects and funding cycles.

### 4.1 Architectural decisions

The work focused on technical and semantic barriers, with maintenance and extensibility costs as the primary drivers of unsustainable research infrastructure. Organizational barriers such as ethics approvals and data use agreements, together with institutional governance more broadly, fall outside the scope of this work. Similarly, full integration of clinical and research documentation through openEHR-based forms requires a hospital information system that operates natively on openEHR, a condition not met at Charité. In the current deployment, study-specific prospective capture eliminates the ETL step for research variables, but not the parallel documentation for routine clinical data. The five studies were non interventional. For trials under clinical trial regulation, a validated electronic data capture system is the system of record. An openEHR forms system can be validated to that level like any other, but this environment has not been. Where the hospital system itself runs on openEHR, study fields could be captured within primary documentation, as additional fields in the clinical forms the staff already use. This has the potential of making the eCRF a component of the HIS itself instead of a separate system.

A CDR addresses fragmentation in both retrospective and prospective data, serving as a single governed persistence layer into which prospective data is captured directly through standard-based forms (35) and from which openEHR, FHIR, OMOP, and future representations are derived on demand. In the institutional context of this deployment, FHIR, OMOP, and openEHR exist as targets but none serves as a governed semantic layer, and the same source data is independently transformed into heterogeneous structures across over 200 extraction requests (26). The 200 extraction requests observed in the institutional context illustrate the cost trajectory of treating standards as exchange targets rather than persistence layers. Each request reproduces transformation logic that a governed CDR amortizes once. The CDR choice therefore reflects where semantic governance is most efficiently enforced, not a preference for openEHR itself.

### 4.2 Archetype governance and knowledge reuse

The five studies span multiple clinical domains and funding sources, selected to exercise the architecture across heterogeneous use cases rather than to optimize for archetype reuse. Across the five studies, 57 of 125 archetypes were shared between at least two studies, demonstrating reuse within the deployment. Approximately a third of archetypes were defined locally, representing Germany-specific concepts and demographic models not suited to international normalization, alongside gaps in the international library tagged for CKM submission. The international submission and review process demands time and resources that individual projects rarely budget for, and governance contributions tend to lag behind modeling needs. Despite this overhead, the process amortizes across the community at a scale that project-local approaches cannot achieve. Where archetypes addressed overarching use cases, review was conducted within the HiGHmed consortium, with internal review applied for locally scoped models in the interim. The 68.8 percent international reuse figure carries an important caveat for transferability. It reflects the cumulative archetype work of the international community at the time of this deployment, and institutions starting today benefit from a larger published library. Those addressing genuinely novel clinical domains, by contrast, will see lower initial reuse and depend on local modeling until community contributions catch up. The reuse figure is therefore a function of the community’s accumulated work, not a property of the architecture itself. Operationally, the process has three tracks. Archetypes follow the governance of the CKM, local refinement, upload and review rounds with clinical input until consensus (29). Archetypes that serve only local needs, such as institutional identifiers, stay local without upload. Templates are designed per study and iterated within the team and, where a template is shared, with the other sites until all agree. Mappings are written per archetype, peer reviewed and tested within the team, uploaded to the public repository where no intellectual property constraints apply, and reviewed by the community (7, 32). The skills required are a mix of technical modelling and clinical domain understanding, with access to clinicians for consensus either locally or through the community, for instance on the openEHR discourse forum. Thoroughness is needed as well, since whether a field carries the semantics a study needs is often a matter of judgement.

The archetypes, their governance history, and the transformation mappings built on them are a reusable asset that outlasts the technical stack and underwrites longitudinal semantic stability across studies. The contribution is this treatment of the mappings as versioned and reviewed infrastructure, not the choice of openEHR, FHIR and OMOP, which are the standards the mappings currently connect. The deployment maturation observed across studies is consistent with this property. The team observed that each new study required less adaptation effort than its predecessor, and transitions between clinical domains required changes at the archetype and template level only, with the infrastructure components reused. The reduction reflects reuse at two levels, the community library that supplies most archetypes and half of the mappings of every study, and the platform’s own assets, which a study in a covered domain such as ACRIBIS takes over almost completely. The architectural separation of clinical knowledge from the technical reference model enables this transfer between clinically distinct domains. Team familiarity with the platform also accumulates but contributes less directly, since each new clinician group requires fresh template review.

A direct comparison against the institutional data lake is not feasible without duplicating study execution across both systems, and infrastructure contributions of this kind are evaluated through operational evidence rather than controlled trials (36, 37). The two systems are also optimized for different goals. The data lake offers near-complete coverage of raw data at the cost of structure, so each secondary use re-derives semantics from source exports and little of that effort transfers between projects. The CDR inverts this trade-off, paying modelling cost once per clinical domain in exchange for reusable querying, transformation and export. The limited patient coverage reported here is therefore a consequence of that design rather than a deficiency of it, and the two approaches remain complementary.

Within that scope, the environment runs across five studies spanning multiple clinical domains and funding sources, integrates heterogeneous data types under a shared semantic model, and addresses the structural challenges documented. The evidence presented here nonetheless reflects a single primary deployment institution, and broader generalizability awaits adoption in institutions with different EHR landscapes and governance contexts. The evidence is descriptive. What was measured is the origin of the artifacts of each study and the staffing that operates the environment. Implementation time, maintenance time and system performance were not recorded. Sustainability is the goal the architecture is designed for, and what five studies can show is that the artifacts of one study served the next. Whether the environment stays sustainable across further funding cycles is a question this deployment cannot yet answer. Federation across distributed CDR instances is currently demonstrated at three validation nodes but has not yet been used in production, and extending it to routine operation remains future work. openEHR also has less mature tooling for secondary use than the OHDSI ecosystem, currently mitigated by projecting data into the OMOP representation via Eos for analytics. Building richer native openEHR tooling is similarly future work.

### 4.3 Team and operating effort

The environment is developed and operated by two research associates and a team lead, who also carry out the studies, so none of the three works on the environment full time. They built the archetypes and templates, the study forms, the ingestion pipelines and the FHIR and OMOP mappings. One administrator from the institutional IT department sets up and updates the CDR and helped with some ETL pipelines, and a support contract with the CDR vendor covers bug fixes and tickets. None of the five studies has yet requested a FHIR or OMOP export, so neither pipeline is operated in production at Charité, and running them in production would add validation and monitoring effort that the current staffing does not include. Their specifications and software are maintained jointly with other institutions in community working groups (7, 32). The effort grows with new source systems and clinical domains, not with the number of studies.

### 4.4 Comparison to related work

Haarbrandt et al. proposed the HiGHmed open platform as a consortium-level architecture combining openEHR-based data integration centers with governed clinical knowledge models across multiple institutions (36), articulating design principles for open-platform research infrastructure without evaluating an institutional deployment or its sustainability. Pedrera-Jiménez et al. propose an agnostic framework for combining openEHR, ISO 13606, and FHIR within a shared health data environment, addressing standards compatibility, not the design or deployment of a sustainable research infrastructure (37). Prokosch et al. (38) and Lenert et al. (39) demonstrated CDR-centered pipelines in production multi-site deployments, but both are scoped to a single disease context under pandemic-driven conditions and neither addresses the governance and reusability requirements of heterogeneous, independently funded research programs.

Schreiweis et al. (40) report an openEHR-based health knowledge management platform, derived from a structured requirements engineering process. Their focus is the operational integration of patient care and research within a single institution, evaluated through cardiology and radiology workflows including cohort identification and molecular tumor board support. This is complementary to the present work, which targets long-term maintainability and governance across studies and funding cycles, motivating the emphasis on archetype reuse and reusable transformation pipelines released as a deployable open-source stack.

### 4.5 Alignment with the European health data space

The European Health Data Space (EHDS) (15) fixes obligations for data holders while leaving the technical formats to implementing acts expected from 2027. Clinical data is governed at the persistence layer in international clinical models that are independent of any exchange format, so the format the implementing acts select becomes one more projection from the same repository, as FHIR and OMOP are today. The current demonstrators for the European Electronic Health Record Exchange Format (EEHRxF) build on FHIR, and FHIRconnect mappings towards them are in progress. The environment is itself part of the i2x demonstrator project testing the EEHRxF, whose ETL pipelines and related components were still being built at the time of submission and which is therefore not among the studies reported here.

### 4.6 Outlook

The broader relevance of this work extends beyond the deployment site and beyond any specific standard. Governing data at the persistence layer is intended to make downstream use sustainable, whether for analytics or for federated and AI-driven research. Longitudinal patient records spanning routine care, prospective capture and other modalities become queryable under a shared semantic model that supports federation without centralization and produces AI-ready datasets as a product of infrastructure, not per-project preparation. openEHR is a standard for standardizing the EHR. Mapping research data into it turns secondary use into a driver of primary use standardization.

## 5 Conclusion

Fragmented, project-driven research infrastructures accumulate technical debt that ad-hoc solutions defer rather than resolve. The architecture presented here addresses this at the structural level by establishing a semantically governed clinical data repository as the shared persistence layer for multiple studies, from which standardized FHIR and OMOP representations can be derived without per-project reimplementation. Deployed at Charité across five studies and approximately 16,000 patients, the environment shows that reusable research infrastructure is achievable within an existing healthcare IT landscape. Governed archetypes and version-controlled pipelines carried the semantic foundation from one study to the next, converting what would otherwise accumulate as institutional debt into a shared, reusable asset. Prospective data capture through openEHR-based forms avoids a separate electronic data capture system for research variables. Removing duplicate documentation with routine care additionally requires a hospital information system that runs on openEHR, which Charité does not have, so parallel documentation remained in this deployment. Federation nodes tested at three institutions show that queries can run across sites that share the same models, while routine multicentre operation has not yet been reached. The open-source tooling and deployable configuration lower the barrier for adoption at other institutions.

## Data Availability

All software developed in this work, including the deployable Helm chart and Docker Compose configuration, is publicly available on GitHub at https://github.com/AltarisV/OHS-Open-Health-Stack. The clinical datasets are not publicly available due to data protection regulations and patient privacy.
