# Supplementary material for: An interoperable and reusable research environment for digital medicine
Source: Front Digit Health. 2026 Oct 28;8:1943167. doi: 10.3389/fdgth.2026.1943167 (PMC13752112; doi:10.3389/fdgth.2026.1943167)
Supplement: Supplementary file 1 [file Supplementaryfile1.pdf]

Supplementary Material

Supplementary Table S1 | Mapping potential across studies, in the order in which the studies came onto the platform. Mappable archetypes are all archetypes except compositions and sections, which carry no mapping. Shipped library counts the archetypes covered by the OMOCL and FHIRconnect libraries as shipped, earlier studies those an earlier study would have mapped, still to write the rest, which is added to the pool for the next study. No mapping was written for these studies, the table shows how coverage would have accumulated. Reuse total is the sum of the two covered columns.

Study	Mappable archetypes	Shipped library	Earlier studies	Reuse total	Still to write
OMOP (OMOCL)					
HiGHmed-UCC	62	36 (58%)	0 (0%)	36 (58%)	26 (42%)
CAEHR-C	59	32 (54%)	11 (19%)	43 (73%)	16 (27%)
ETL-AI	13	6 (46%)	1 (8%)	7 (54%)	6 (46%)
CALM-QE	50	27 (54%)	10 (20%)	37 (74%)	13 (26%)
ACRIBIS	24	17 (71%)	7 (29%)	24 (100%)	0 (0%)
FHIR (FHIRconnect model mappings)					
HiGHmed-UCC	62	24 (39%)	0 (0%)	24 (39%)	38 (61%)
CAEHR-C	59	16 (27%)	22 (37%)	38 (64%)	21 (36%)
ETL-AI	13	7 (54%)	1 (8%)	8 (62%)	5 (38%)
CALM-QE	50	14 (28%)	15 (30%)	29 (58%)	21 (42%)
ACRIBIS	24	10 (42%)	13 (54%)	23 (96%)	1 (4%)
